# Classification of a Hypervirulent *Aeromonas hydrophila* Pathotype Responsible for Epidemic Outbreaks in Warm-Water Fishes

**DOI:** 10.3389/fmicb.2016.01615

**Published:** 2016-10-18

**Authors:** Cody R. Rasmussen-Ivey, Mohammad J. Hossain, Sara E. Odom, Jeffery S. Terhune, William G. Hemstreet, Craig A. Shoemaker, Dunhua Zhang, De-Hai Xu, Matt J. Griffin, Yong-Jie Liu, Maria J. Figueras, Scott R. Santos, Joseph C. Newton, Mark R. Liles

**Affiliations:** ^1^Department of Biological Sciences, Auburn UniversityAuburn, AL, USA; ^2^School of Fisheries, Aquaculture and Aquatic SciencesAuburn, AL, USA; ^3^Alabama Fish Farming CenterGreensboro, AL, USA; ^4^Aquatic Animal Health Research Unit, United States Department of Agriculture-Agricultural Research ServiceAuburn, AL, USA; ^5^Thad Cochran National Warmwater Aquaculture Center, College of Veterinary Medicine, Mississippi State UniversityStoneville, MS, USA; ^6^College of Veterinary Medicine, Nanjing Agricultural UniversityNanjing, China; ^7^Departamento de Ciencias Médicas Básicas, Facultad de Medicina y Ciencias de la Salud, IISPV, Universidad Rovira i VirgiliReus, Spain; ^8^Department of Pathobiology, Auburn UniversityAuburn, AL, USA

**Keywords:** *Aeromonas hydrophila*, pathogenesis, comparative genomics, emerging disease, bacteria, catfish, carp

## Abstract

Lineages of hypervirulent *Aeromonas hydrophila* (vAh) are the cause of persistent outbreaks of motile *Aeromonas* septicemia in warm-water fishes worldwide. Over the last decade, this virulent lineage of *A. hydrophila* has resulted in annual losses of millions of tons of farmed carp and catfish in the People's Republic of China and the United States (US). Multiple lines of evidence indicate US catfish and Asian carp isolates of *A. hydrophila* affiliated with sequence type 251 (ST251) share a recent common ancestor. To address the genomic context for the putative intercontinental transfer and subsequent geographic spread of this pathogen, we conducted a core genome phylogenetic analysis on 61 *Aeromonas* spp. genomes, of which 40 were affiliated with *A. hydrophila*, with 26 identified as epidemic strains. Phylogenetic analyses indicate all ST251 strains form a coherent lineage affiliated with *A. hydrophila*. Within this lineage, conserved genetic loci unique to *A. hydrophila* were identified, with some genes present in consistently higher copy numbers than in non-epidemic *A. hydrophila* isolates. In addition, results from analyses of representative ST251 isolates support the conclusion that multiple lineages are present within US vAh isolated from Mississippi, whereas vAh isolated from Alabama appear clonal. This is the first report of genomic heterogeneity within US vAh isolates, with some Mississippi isolates showing closer affiliation with the Asian grass carp isolate ZC1 than other vAh isolated in the US. To evaluate the biological significance of the identified heterogeneity, comparative disease challenges were conducted with representatives of different vAh genotypes. These studies revealed that isolate ZC1 yielded significantly lower mortality in channel catfish, relative to Alabama and Mississippi vAh isolates. Like other Asian vAh isolates, the ZC1 lineage contains all core genes for a complete type VI secretion system (T6SS). In contrast, more virulent US isolates retain only remnants of the T6SS (*clpB, hcp, vgrG*, and *vasH*) which may have functional implications. Collectively, these results characterize a hypervirulent *A. hydrophila* pathotype that affects farmed fish on multiple continents.

## Introduction

Aquaculture industries across the world have been decimated by epidemics of a hypervirulent pathotype of *A. hydrophila* (vAh) (Nielsen et al., 2001; Hemstreet, 2010). *A. hydrophila* is ubiquitous within warm-water environments and has a diverse host range (i.e., amphibians, birds, fishes, reptiles, and mammals) with equally diverse diseases that include motile *Aeromonas* septicemia (MAS) in fish. MAS produces significant internal and external hemorrhage and exophthalmia, often followed by mortality within several hours of manifestation of disease (Xu et al., 1993; Camus et al., 1998; Nielsen et al., 2001; da Silva et al., 2012; Beaz-Hidalgo et al., 2013).

The first report of the vAh pathotype was *A. hydrophila* J-1 in 1989, which was isolated from epizootics of MAS in China's Jiangsu Province (Chen and Lu, 1991). This strain was categorized as sequence type 251 (ST251) and recognized as being capable of causing high mortality in grass carp (*Ctenopharyngodon idella*). Outbreaks of MAS in farmed carp have persisted in China, resulting in losses estimated at 2,200 tons of dead fish per year (Chen and Lu, 1991; Nielsen et al., 2001; Zhang et al., 2002; Pang et al., 2015). Epidemics of ST251-associated MAS occurred within the same province in 2010, with vAh isolate *A. hydrophila* NJ-35 identified as the etiologic agent (Pang et al., 2012). Another vAh isolate from China (*A. hydrophila* ZC1) was isolated from grass carp exhibiting signs of hemorrhagic septicemia from an aquaculture farm in China's Guangdong Province (Deng et al., 2009). In general, MAS is a regular occurrence each summer, resulting in significant economic losses in the Chinese aquaculture industry, with estimates exceeding five billion yuan per year (Prof. Hui Chen, personal communication).

In 2004, the first reported case of ST251-related MAS in the US arose when *A. hydrophila* S04-690 was isolated from diseased channel catfish (*Ictalurus punctatus*) from a catfish farm in Washington County, Mississippi (MS) (Hossain et al., 2014). Beginning in 2009, vAh strains were consistently recovered from recurring outbreaks of MAS in aquaculture ponds in western Alabama (AL) with a reported 2,000 tons of dead fish in the first year (Hemstreet, 2010). To date, this number has grown to exceed an estimated 10,500 tons, with vAh isolates representing the largest percentage (35%) of disease cases at the Alabama Fish Farming Center (Hemstreet, 2015). Although representative data on production losses attributed solely to vAh are difficult to attain, vAh clearly represents a significant threat to warm-water aquaculture industries.

Previous comparative genomic analyses of vAh strains isolated from catfish in the US and carp in China indicated these strains share a recent common ancestor (Hossain et al., 2014). Within this monophyletic clade, vAh strains isolated from carp and catfish have unique phenotypes and genotypes (L-fucose metabolism, an inducible prophage and the ability to use *myo*-inositol as a sole carbon source) that distinguish them from more typical strains of *A. hydrophila* not associated with epizootics (non-vAh) (Hossain et al., 2013, 2014; Pang et al., 2015). These studies also demonstrated that MS vAh strain S04-690 is more similar to the carp isolate ZC1 than to other vAh strains from AL (Hossain et al., 2014). Since this study was published, MAS outbreaks attributed to vAh have spread to the Delta region of west Mississippi, with anecdotal reports from the industry suggesting annual losses in MS now exceed 150 tons. The purpose of this study was to characterize the vAh pathotype by examining vAh strains collected from farmed catfish in AL and MS in recent years and compare genome sequences of all available ST251 strains together with other *Aeromonas* spp. genomes available in GenBank by phylogenomic analysis, determine the presence of putative virulence factors in vAh and non-vAh lineages and assess the relative capacity of selected strains to cause MAS in channel catfish.

## Methods

### Bacterial strains–disease isolates and catfish challenge

*A. hydrophila* isolates for disease challenge were recovered from diseased channel catfish from commercial aquaculture operations in western Alabama and the Delta region of west Mississippi. Briefly, catfish demonstrating symptoms typical of MAS were collected in a moribund state and submitted for diagnostic evaluation and necropsy at Auburn University in Auburn, AL or Mississippi State University's Aquatic Research and Diagnostic Laboratory at the Thad Cochran National Warmwater Aquaculture Center (NWAC) in Stoneville, MS. Liver and kidney tissues were sampled for aerobic bacterial cultures. Samples of tissue were homogenized in sterile phosphate buffered saline and portions of the homogenate streaked onto tryptic soy agar (TSA; Beckton Dickinson, Franklin Lakes, NJ) or brain heart infusion (BHI; Beckton Dickinson) for bacterial isolation. Pure cultures were identified as vAh strains by the vAh-specific qPCR method previously described and/or utilization of *myo-*inositol as a sole carbon source (Griffin et al., 2013; Hanson et al., 2014). From this sampling design, *A. hydrophila* isolates ML09-119, ML10-51K, S04-690, S14-296, and S14-452 were cryogenically preserved (mixed with 50% glycerol, stored at −80°C) and subsequently used in catfish immersion challenges.

### Infection and histopathology of channel catfish using an immersion challenge model

Channel catfish were obtained as fry from the USDA-ARS Warmwater Aquaculture Research Unit housed at NWAC and reared to experimental size in 340 liter troughs supplied with 26 ± 2°C dechlorinated municipal water under pathogen-free conditions. All animal experiments were approved by, and conducted in compliance with, regulations of the Institutional Animal Care and Use Committee of the Aquatic Animal Health Research Unit (USDA-ARS) in Auburn, Alabama. Water temperature was maintained at 26 ± 2°C with a centralized heater. Prior to trials, heart, liver, head kidney, trunk kidney, spleen, brain, and skeletal muscle tissues were collected from 10 randomly sampled fish and sampled by culturing on BHI to verify fish were not presently infected with vAh.

Two hundred catfish fingerlings, with a mean weight of 111 ± 47 g and length of 19 ± 3 cm, were acclimated for 12 days in 56-L glass aquaria (10 fish per tank, 3 tanks per isolate, and 2 mock infected control tanks) containing about 50-L water prior to challenge. The immersion challenge was conducted using the recently described fin clip method (Zhang et al., 2016). At the time of infection, water volume was reduced to 15-L per tank. To sedate animals for handling, fish were netted from individual aquaria and placed into a container filled with 20-L of dechlorinated water containing 150 mg/L of buffered Tricaine-S (tricaine methanesulfonate; Western Chemical, Inc., Ferndale, WA). Once fish were anesthetized, the adipose fin was clipped at its base and fish were returned to respective aquaria for recovery from anesthesia.

For the bacterial challenge, 100 mL of tryptic soy broth (TSB) containing approximately 3.0 × 10^9^ CFU/mL of the respective *A. hydrophila* strains (ML-09-119, ML10-51K, S04-690, S14-296, S14-452, and ZC1) was added to each of three aquaria, resulting in an approximate challenge dose of ~2.0 × 10^7^ CFU/mL. Two tanks served as mock infected controls, receiving only 100 mL sterile TSB. After 1-h exposure, water flow to aquaria (0.5 L/min) was resumed. Fish mortality was monitored daily for 7 days. At least 50% of dead fish were sampled for confirmation for the presence of vAh in liver and kidney tissues using M9 minimal medium containing 0.3% (w/v) *myo*-inositol (M9I) agar (Hanson et al., 2014). Moribund fish were removed from aquaria daily and surviving fish were euthanized by at least 15 min exposure to 300 mg/L buffered Tricaine-S solution, then necropsied. Heart, liver, head kidney, trunk kidney, spleen, brain, and skeletal muscle tissues were harvested and fixed in 10% buffered formalin for histopathology. Tissues were also collected and used for bacterial identification and quantitation. Formalin fixed tissues were processed and embedded in paraffin. The tissues were cut in four micron sections, stained with hematoxylin and eosin and evaluated for microscopic lesions by light microscopy.

### Bacterial strains–comparative genomics

In total, 61 complete and draft *Aeromonas* spp. genomes were included in this study (Table 1) which included *A. hydrophila* representative genomes as well as *Aeromonas* spp. genomes that are now recognized as having had an erroneous affiliation with *A. hydrophila* (Figueras et al., 2014; Beaz-Hidalgo et al., 2015a). These genomes were retrieved from the US National Center for Biotechnology Information (NCBI) GenBank database and included two *A. caviae* isolates, one *A. dhakensis* isolate, one *A. enteropelogenes* isolate, one *A. media* isolate, one *A. molluscorum* isolate, one *A. taiwanensis* isolate, one *Aeromonas* sp. isolate, 26 *A. hydrophila* fish vAh disease isolates, and 28 non-vAh *A. hydrophila* isolates.

**Table 1:** **Bacterial genomes used in comparative genomic analyses**.

**Strain**	**Phenotype**	**Isolation source**	**GenBank species assignation**	**Species based on phylogeny and ANI**	**Accession**	**Reference**
Ae398	Non-vAh	Human	*A. caviae*	*A. caviae*	SAMEA2272404	Beatson et al., 2011
YL12	Non-vAh	Compost	*A. caviae*	*A. caviae*	SAMN02870964	Lim et al., 2014
AAK1	Non-vAh	Clinical	*A. dhakensis*	*A. dhakensis*	SAMD00036618	Martínez-Murcía et al., 2008
1999lcr	Non-vAh	Clinical	*A. trota*	*A. molluscorum*	SAMN02732394	Dallagassa et al., Unpublished
116	Non-vAh	Clinical	*A. hydrophila*	*A. dhakensis*	NZ_ANPN00000000.1	Chan et al., 2011
14	Non-vAh	Clinical	*A. hydrophila*	*A. dhakensis*	NZ_AOBM00000000.1	Chan et al., 2011
173	Non-vAh	Clinical	*A. hydrophila*	*A. dhakensis*	NZ_AOBN00000000.1	Chan et al., 2011
187	Non-vAh	Clinical	*A. hydrophila*	*A. dhakensis*	NZ_AOBO00000000.1	Chan et al., 2011
226	Non-vAh	Clinical	*A. hydrophila*	*A. hydrophila*	NZ_JEML00000000.1	Chan et al., 2011
259	Non-vAh	Clinical	*A. hydrophila*	*A. dhakensis*	NZ_AOBP00000000.1	Chan et al., 2011
277	Non-vAh	Clinical	*A. hydrophila*	*A. dhakensis*	NZ_AOBQ00000000.1	Chan et al., 2011
4AK4	Non-vAh	Industrial	*A. hydrophila*	*A.* sp. nov.	NZ_CP006579.1	Gao et al., 2013
AD9	Non-vAh	Soil	*A. hydrophila*	*A. hydrophila*	NZ_JFJO00000000.1	Lenneman and Barney, 2014
Ae34	Non-vAh	Koi carp	*A. hydrophila*	*A. hydrophila*	NZ_BAXY00000000.1	Jagoda et al., 2014
AH10	Non-vAh	Grass carp	*A. hydrophila*	*A. hydrophila*	NZ_CP011100.1	Xu et al., 2013
AL06-01	Non-vAh	Bluegill	*A. hydrophila*	*A. hydrophila*	SAMN01085623	Hossain et al., 2013
AL06-06	Non-vAh	Goldfish	*A. hydrophila*	*A. hydrophila*	NZ_CP010947.1	Tekedar et al., 2015
AL10-121	Non-vAh	Channel catfish	*A. hydrophila*	*A. hydrophila*	NZ_LRRW00000000.1	Hossain, 2012
AL97-91	Non-vAh	Channel catfish	*A. hydrophila*	*A. hydrophila*	SAMN04967787	Hossain, 2012
ATCC7966T	Non-vAh	Milk tin	*A. hydrophila*	*A. hydrophila*	NC_008570.1	Seshadri et al., 2006
BWH65	Non-vAh	Clinical	*A. hydrophila*	*A. caviae*	NZ_LESK00000000.1	Earl et al., 2015
E1	Non-vAh	Clinical	*A. hydrophila*	*A. hydrophila*	SAMN01886638	Grim et al., 2013
E2	Non-vAh	Clinical	*A. hydrophila*	*A. hydrophila*	SAMN01886639	Grim et al., 2013
GA97-22	Non-vAh	Rainbow trout	*A. hydrophila*	*Aeromonas* spp.	SAMN01085627	Hossain, 2012
HZM	Non-vAh	Soil	*A. hydrophila*	*A. caviae*	SAMN02596469	Chua et al., 2015
KOR1	Non-vAh	Mangrove	*A. hydrophila*	*A. dhakensis*	NZ_LJOE00000000.1	Yin et al., 2015
MN98-04	Non-vAh	Tilapia	*A. hydrophila*	*A. hydrophila*	SAMN04967900	Hossain, 2012
RB-AH	Non-vAh	Soil	*A. hydrophila*	*A. hydrophila*	NZ_JPEH00000000.1	Rheault et al., 2015
S14-230	Non-vAh	Tilapia	*A. hydrophila*	*A. hydrophila*	SAMN05292364	This study
SSU	Non-vAh	Clinical	*A. hydrophila*	*A. dhakensis*	NZ_AGWR00000000.1	Ribeiro et al., 2012
TN97-08	Non-vAh	Bluegill	*A. hydrophila*	*A. hydrophila*	NZ_LNUR00000000.1	Hossain, 2012
YL17	Non-vAh	Compost	*A. hydrophila*	*A. dhakensis*	NZ_CP007518.2	Lim et al., 2016
WS	Non-vAh	Water sample	*A. media*	*A. media*	SAMN02472129	Chai et al., 2012
848T	Non-vAh	Wedge-shells	*A. molluscorum*	*A. molluscorum*	SAMN02471397	Spataro et al., 2013
LMG24683T	Non-vAh	Unknown	*A. taiwanensis*	*A. taiwanensis*	SAMEA2752407	Colston et al., 2014
MDS8	Non-vAh	Dairy sludge	*Aeromonas* sp.	*A. dhakensis*	SAMN02472124	Raychaudhuri et al., 2013
Ahy_Idx71	vAh	Channel catfish	*A. hydrophila*	*A. hydrophila*	SAMN05292361	This study
AL09-71	vAh	Channel catfish	*A. hydrophila*	*A. hydrophila*	NZ_CP007566.1	Pridgeon et al., 2014
AL09-79	vAh	Channel catfish	*A. hydrophila*	*A. hydrophila*	NZ_LRRV00000000.1	Hossain, 2012
ALG15-098	vAh	Channel catfish	*A. hydrophila*	*A. hydrophila*	SAMN05223361	This study
IPRS15-28	vAh	Channel catfish	*A. hydrophila*	*A. hydrophila*	SAMN05223362	This study
J-1	vAh	Crucian carp	*A. hydrophila*	*A. hydrophila*	NZ_CP006883.1	Pang et al., 2015
JBN2301	vAh	Crucian carp	*A. hydrophila*	*A. hydrophila*	NZ_CP013178.1	Yang et al., 2016
ML09-119	vAh	Channel catfish	*A. hydrophila*	*A. hydrophila*	NC_021290.1	Liles et al., 2011
ML09-121	vAh	Channel catfish	*A. hydrophila*	*A. hydrophila*	NZ_LRRX00000000.1	Hossain, 2012
ML09-122	vAh	Channel catfish	*A. hydrophila*	*A. hydrophila*	NZ_LRRY00000000.	Hossain, 2012
ML10-51K	vAh	Channel catfish	*A. hydrophila*	*A. hydrophila*	SAMN05223363	This study
NJ-35	vAh	Crucian carp	*A. hydrophila*	*A. hydrophila*	NZ_CP006870.1	Pang et al., 2015
PB10-118	vAh	Channel catfish	*A. hydrophila*	*A. hydrophila*	SAMN01085622	Hossain, 2012
pc104A	vAh	Soil	*A. hydrophila*	*A. hydrophila*	NZ_CP007576.1	Pridgeon et al., 2014
S04-690	vAh	Channel catfish	*A. hydrophila*	*A. hydrophila*	SAMN02404466	Hossain et al., 2014
S13-612	vAh	Channel catfish	*A. hydrophila*	*A. hydrophila*	SAMN05292362	This study
S13-700	vAh	Channel catfish	*A. hydrophila*	*A. hydrophila*	SAMN05292363	This study
S14-296	vAh	Channel catfish	*A. hydrophila*	*A. hydrophila*	SAMN05292365	This study
S14-452	vAh	Channel catfish	*A. hydrophila*	*A. hydrophila*	SAMN05256776	This study
S14-458	vAh	Channel catfish	*A. hydrophila*	*A. hydrophila*	SAMN05223364	This study
S14-606	vAh	Channel catfish	*A. hydrophila*	*A. hydrophila*	SAMN05292366	This study
S15-130	vAh	Channel catfish	*A. hydrophila*	*A. hydrophila*	SAMN05223365	This study
S15-242	vAh	Channel catfish	*A. hydrophila*	*A. hydrophila*	SAMN05223366	This study
S15-400	vAh	Channel catfish	*A. hydrophila*	*A. hydrophila*	SAMN05223367	This study
S15-591	vAh	Channel catfish	*A. hydrophila*	*A. hydrophila*	SAMN05223368	This study
ZC1	vAh	Grass carp	*A. hydrophila*	*A. hydrophila*	SAMN02404465	Hossain et al., 2014

**Figure 1:**
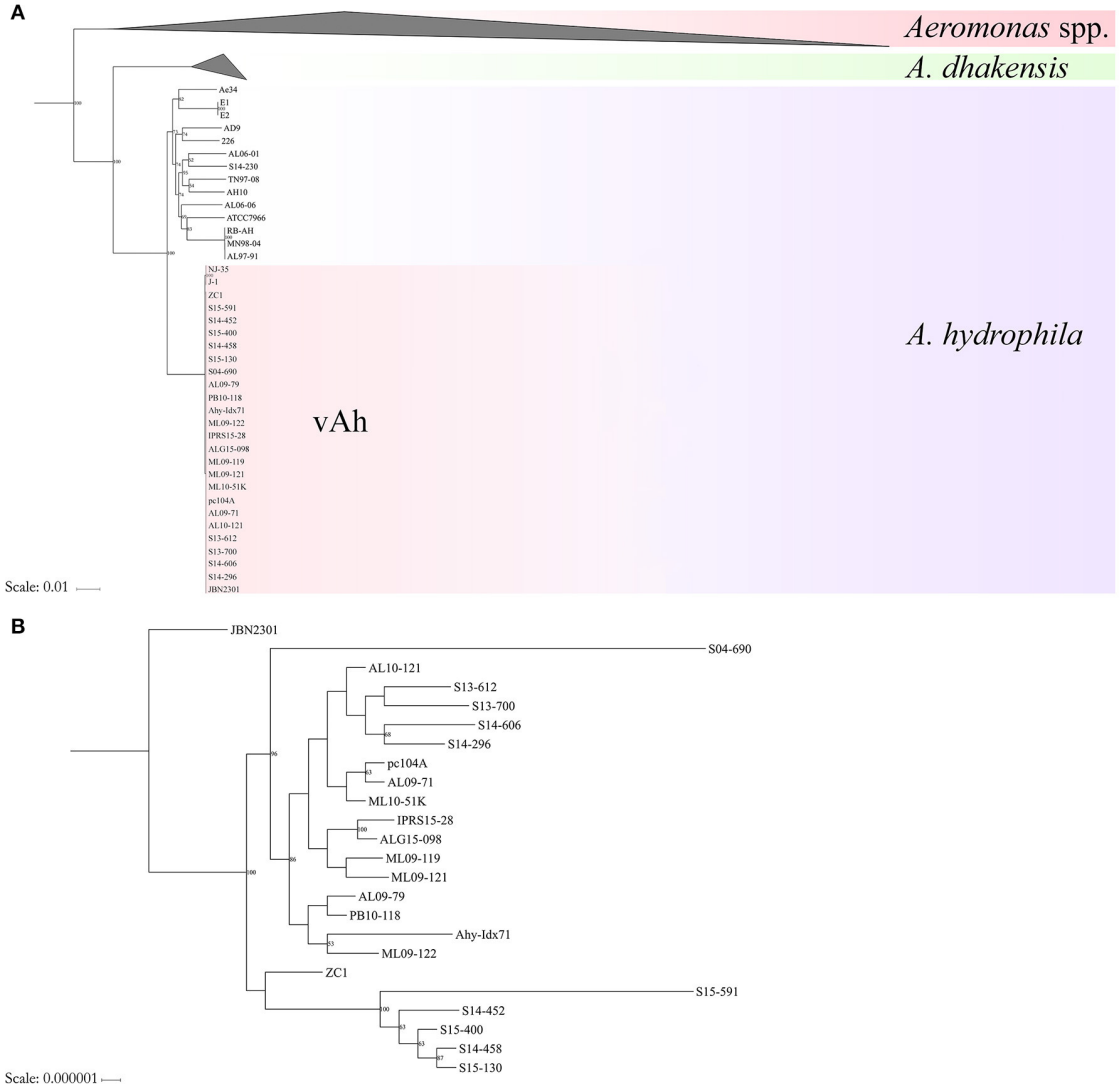
**Maximum likelihood (ML) phylogeny of (Panel A) *Aeromonas* spp. and (Panel B) vAh isolates based on the core genome of 3.78 Mb conserved among these bacterial strains**.

Strains are indicated as virulent A. hydrophila (vAh) or other Aeromonas spp. based on their phylogenetic affiliation (Figure 1).

### Genome sequencing

Strains representative of different vAh lineages were selected for Illumina sequencing based on results of vAh genotype-specific PCR (see below) from a total of 38 suspected vAh isolates recovered from diagnostic cases in MS between 2013 and 2015. Strains were selected so that isolates represented MAS outbreaks from multiple geographically discrete operations. Genome sequencing with 250 bp read-length using paired-end sequencing was performed on the Illumina MiSeq platform using the Nextera XT kit (Illumina, San Diego, CA) to prepare bar-coded fragment libraries according to the manufacturer's protocol. Sequence reads were trimmed and quality sequence reads were assembled *de novo* using the CLC Genomics Workbench (Qiagen, Redwood City, CA) using default settings. vAh strain draft genomes (*n* = 14) were generated for strains Ahy_Idx7_1, ALG15-098, IPRS-15-28, ML10-51K, S13-612, S13-700, S14-230, S14-296, S14-458, S14-606, S15-130, S15-242, S15-400, and S15-591 (Supplemental Table 1). In addition to a standard Illumina MiSeq run for vAh strain S14-452, a NxSeq 20 kb mate pair library was constructed and sequenced using an Illumina MiSeq at the Lucigen Corporation (Middleton, WI). The *de novo* assembly from the standard Illumina MiSeq sequences resulted in 13 contigs (80.15 average coverage) whereas the combination of these sequences together with the mate pair-derived sequences using *de novo* assembly with SPAdes (v3.5.0) resulted in a complete genome sequence.

### Pathotype-specific PCR for vAh genotypes

Evaluation of the previously described vAh-specific qPCR primer set (listed as 2986L and 2986R in Table 2) (Griffin et al., 2013) was performed *in silico* using Geneious v. R9 with a maximum mismatch setting of two bases and a band prediction interval of between 100 and 1000 bases, which predicted that numerous vAh isolates (Ahy_Idx7_1, J-1, AL09-79, AL10-121, JBN2301, ML09-121, ML09-122, NJ-35, PB10-118, S13-612, and S15-591) would not produce an amplicon. To address this potential pitfall, we evaluated the primer set using touchdown PCR on all available and relevant vAh and non-vAh isolates (data not shown). Touchdown PCR was performed on an Eppendorf Mastercycler Gradient S with 50 ng of template gDNA extracted from each isolate (E.Z.N.A.® Bacterial DNA Kit; Omega Biotek, Georgia, USA), 13 μl of Econotaq Plus Green 2x MasterMix (Lucigen, Madison, Wisconsin, USA), and 20 picomoles of each primer in a 25-μl reaction. Cycling parameters comprised of an initial denaturation of 94°C for 3 min; 10 cycles of 94°C for 30 s, 68°C for 30 s (−1^o^C per cycle), and 72°C for 1 min; followed by 25 cycles of 94°C for 30 s, 58°C for 30 s, and 72°C for 1 min; and a final extension at 72°C for 5 min. Amplicons were resolved on a 1% agarose gel and stained with ethidium bromide.

**Table 2:** **Oligonucleotide primers specific to members of the vAh pathotype (vAh-SerF and vAh-SerR), previously described qPCR vAh primers (2986F and 2986R), and primers used to screen for unique isolates used in this study (ML09-119F, ML09-119R, S14-452F, S14-452R, ZC1F, and ZC1R)**.

**Primer name**	**Direction**	**Sequence**	**Amplicon size (bp)**
vAh-SerF	Forward	5′-AG′CATCACCAGCGTTGGCCC-3′	502
vAh-SerR	Reverse	5′-GCCGGGCTGAACTTCCGCAT-3′	
2986F	Forward	5′-CTATTACTGCCCCCTCGTTC-3′	167
2986R	Reverse	5′-ATTGAGCGGTATGCTGTCG-3′	
ML09-119F	Forward	5′-GTTCCGTTCCATCTGTTCGTGA-3′	246
ML09-119R	Reverse	5′-CAACCATCTTGGTCGCAATC-3′	
S14-452F	Forward	5′-CAGAACGTGCTGCAGAGATTGA-3′	350
S14-452R	Reverse	5′-TCCGAGAATTCGATGACGAAGG-3′	
ZC1F	Forward	5′-GCAATTCTGCGGTCACTTCTCG-3′	400
ZC1R	Reverse	5′-AGCGTACCGTCTCGTCGATATG-3′	

In order to have a PCR assay that differentiates vAh from non-vAh strains while encompassing both US and Asian vAh isolates, new vAh genotype-specific primers (listed as vAh-SerF and vAh-SerR in Table 2) were designed using comparative analysis of annotations produced by the Rapid Annotations using Subsystems Technology (RAST) v2.0 server. These primers target a serine protease gene sequence and are predicted to produce a 502 bp amplicon (data not shown). Touchdown PCR conditions and validation methods were identical to those described previously.

In addition to primers that help to identify vAh strains, *A. hydrophila* lineage-specific primers were developed that were specific to vAh lineages represented by strains ML09-119 (NC_021290), S14-452 (SAMN05256776), and ZC1 (SAMN02404465) (Table 2). The ML09-119 lineage-specific primer set targets the *tnsA* endonuclease gene and produces a 246 bp amplicon, the S14-452 lineage-specific primer set targets the COG3339 genetic locus and produces a 350 bp amplicon, and the ZC1 lineage-specific primer set targets a hypothetical protein and produces a 400 bp amplicon.

To determine the vAh genotype affiliation of disease isolates in AL and MS, genomic DNA was isolated from each isolate (Gentra Puregene DNA isolation kit; Qiagen, Hilden, Germany) and used as a template in a 25-μl PCR that comprised of 13 μl of Econotaq Plus Green 2x MasterMix (Lucigen, Madison, Wisconsin, USA), 20 picomoles of each oligonucleotide primer and 50 ng of template gDNA. Samples were run on a C1000 Touch thermal cycler (BioRad, Hercules, California, USA) with an initial denaturation of 94°C for 3 min followed by 35 cycles of 94°C for 30 s, 58°C for 30 s, and 72°C for 1 min, with a final extension at 72°C for 5 min and amplicon verification was performed as previously described.

### Core genome analyses

A core genome was created using both coding and non-coding sequences of 61 genomes labeled as *A. hydrophila* within GenBank, some of which were revealed to be misclassified. Specifically, any contigs less than 10 Kbp in size were first filtered from draft genomes to increase computational efficiency and decrease false positives by limiting the mapping of smaller fragments to non-homologous and/or multiple regions. In general, smaller fragments contribute less to the production of core genomes as a function of being flanked by areas of heavily repetitive sequences. In the assembly stage, these regions stopped growing and remained short. While core genomes produced by this method are often smaller, the removal of smaller contigs did not compromise the ability to form a phylogenetically-informative core genome.

Filtered sequence data were then submitted as FASTA files to the multiple whole genome alignment tool Mugsy v1.2.3 (Angiuoli and Salzberg, 2011) under default parameters. The resulting alignment was subsequently processed with GBLOCK v0.91b (Castresana, 2000) in order to identify regions of high conservation across all isolates. Parameters for retention by GBLOCK are dictated by the input alignment and were: A minimum of 31 and 51 sequences for conserved and flanked positions, respectively, a maximum of 8 contiguous, but non-conserved positions, a minimal block length of 10, and one-half of the sequences were allowed to possess gapped positions within a block. From the final alignment, a maximum likelihood (ML) phylogeny for the 61 *Aeromonas* spp. isolates, including 54 isolates labeled in GenBank as *A. hydrophila*, was inferred using RAxML v8.2.8 (Stamatakis, 2014) under the General Time Reversible model of evolution with estimated proportions of invariable sites and rate variation among sites (i.e., GTR+I+G) and 1000 bootstrap replicates to determine branch supports. Trees were visualized using Archaeopteryx v.beta 0.9901.

Following generation of a consensus sequence, the National Microbial Pathogen Data Resource (NMPDR) Rapid Annotations using Subsystems Technology (RAST) v2.0 server was used in conjunction with the SEED v2.0 algorithm to annotate the core genome and generate metabolic models (Aziz et al., 2008; Overbeek et al., 2014). These predictive models were evaluated using both protein-protein Basic Local Alignment Search Tool (e.g., BLASTp and BLASTx) algorithms through GenBank as well as the Joint Genome Institute's Genomes OnLine Database (GOLD) v5.0.

### Calculating average nucleotide identity

To assess overall genetic similarity, the average nucleotide identity (ANI) comparison of 61 *Aeromonas* spp. genomes was evaluated using JSpecies (v1.2.1) and cross-validated with the Konstantinidis lab ANI calculator (Richter and Rosselló-Móra, 2009; Rodriguez and Konstantinidis, 2014). According to criteria used for the genus *Aeromonas*, ANI-values >96% indicated strains belong to the same species (Colston et al., 2014; Beaz-Hidalgo et al., 2015a).

### vAh differential gene identification

To evaluate differences in gene content among the 61 genomes, data from the PAthogen Resource Integration Center (PATRIC) protein family sorter tool, RAST/SEED gene annotations, the Pathogen Host Interaction database (http://www.phi-base.org), and the Virulence Factors of Pathogenic Bacteria databases (http://www.mgc.ac.cn/VFs) were combined with copy number data for previously identified virulence genes (Rasmussen-Ivey et al., 2016). Results were evaluated by comparing predicted virulence-associated genes with closely and disparately related isolates to identify genes that were differentially present. Notably, genes shared between vAh and non-vAh isolates or those unique to an individual strain were removed from downstream analyses. These data were subsequently coupled with screening of virulence factors and vAh-associated genes using searches of the NCBI GenBank database with MegaBLAST and BLASTn algorithms (Wheeler et al., 2003). Thresholds for absence were specific to the respective gene and were restricted to mutations altering the predicted functional domains of proteins in which the protein sequence in question returned a functionally divergent protein. Once validated, these gene clusters and virulence factors were transformed for statistical analyses using Orange Data Mining software v.3.3.5 and/or R Studio v.0.99.896. After pre-processing in R Studio using packages MuMIn v.1.15.6, randomForest v.4.6-12, and k-means v.0.1.1, heat maps of resultant data were generated using Orange Data Mining software. To analyze subclade differences, vAh and non-vAh strains with known virulence properties (*n* = 25) were evaluated using k-means at 20 clusters (100% between sum of squares/total sum of squares). In addition to these analyses, the T346 Secretion System Hunter (version is not published) was used to identify T6SS-associated gene clusters using Glimmer v3.02, and HMMER3 v3.1b2 (Martínez-García et al., 2015).

## Results

### Identifying new vAh isolates

The S04-690 genome was previously found to be the genetic intermediate (raw genetic distance) between US catfish vAh isolates (represented by strain ML09-119) and the Asian carp vAh isolate ZC1 (Hossain et al., 2014). A total of 38 vAh isolates recovered from MAS outbreaks in Mississippi from 2013 to 2015 and confirmed as vAh by phenotypic (*myo-*inositol usage) and/or genomic tests (qPCR) (Griffin et al., 2013; Hanson et al., 2014) were subjected to vAh lineage-specific PCR. Testing using lineage-specific primer sets based on representative isolates ML09-119, S14-452, and ZC1 showed that of the 38 MS isolates recovered from MAS outbreaks between 2013 and 2015, 20 isolates from a single farm were positive for the ML09-119 lineage-specific amplicon, while 18 isolates across five geographically discrete farms were positive for the S14-452 lineage-specific amplicon. No isolates produced amplicons with the ZC1 lineage-specific primer set (data not shown). Isolates that produced an amplicon with either the ML09-119 or S14-452 lineage-specific primer sets did not produce amplicons when assayed with other lineage-specific primer sets (data not shown).

### Pathotype-specific PCR for vAh genotypes

Results from touchdown PCR assays demonstrate that the original 2968 primer set (Griffin et al., 2013) reliably distinguishes US vAh from non-vAh isolates (data not shown). However, the Asian carp isolates J-1 and NJ-35 were unable to be experimentally evaluated and *in silico* analysis predicts that Asian isolates J-1 and NJ-35 would not produce a PCR amplicon using the 2968 primer set. To address this possible limitation, a new vAh genotype-specific primer set (listed as vAh-SerF and vAh-SerR in Table 2) was designed to be inclusive of all Asian and US isolates, based on *in silico* analyses of vAh and non-vAh isolates. The vAh-SerF/R primer set was empirically confirmed to produce a 502 bp amplicon in all available US vAh isolates while returning a negative result for non-vAh isolates (data not shown).

### Core genome analysis

Alignment of the complete and filtered draft genomes of the 61 *Aeromonas* spp. genomes via Mugsy produced a matrix of 19,817,762 positions. Following processing with GBLOCK, the core genome of these 61 strains contained 32,401 blocks and a consensus of 3,776,490 bp. This included 120,049 variable (>2 different nucleotides) and 79,507 phylogenetically informative sites (nucleotides that contributed to sorting phylogenetic groups), with percent G+C composition of 62.6%. Notably, these conserved regions collectively have a higher percent G+C content than the 61-isolate average of 61.1% (with a range of 60% for the genome of the type strain of *A. molluscorum* 848^T^ to 63.2% for the one of *A. taiwanensis* LMG24683^T^; *p*-value = 0.003). Across the core analysis, within complete sites, the average transition to transversion ratio was 1.437 for all sequence pairs, with a minimum of 0 transitions and 1 transversion (*A. hydrophila* S04-690 and S13-700) and a maximum of 13 transitions and 2 transversions (*A. hydrophila* 173 and 14).

### Core genome phylogeny

**Figure 2:**
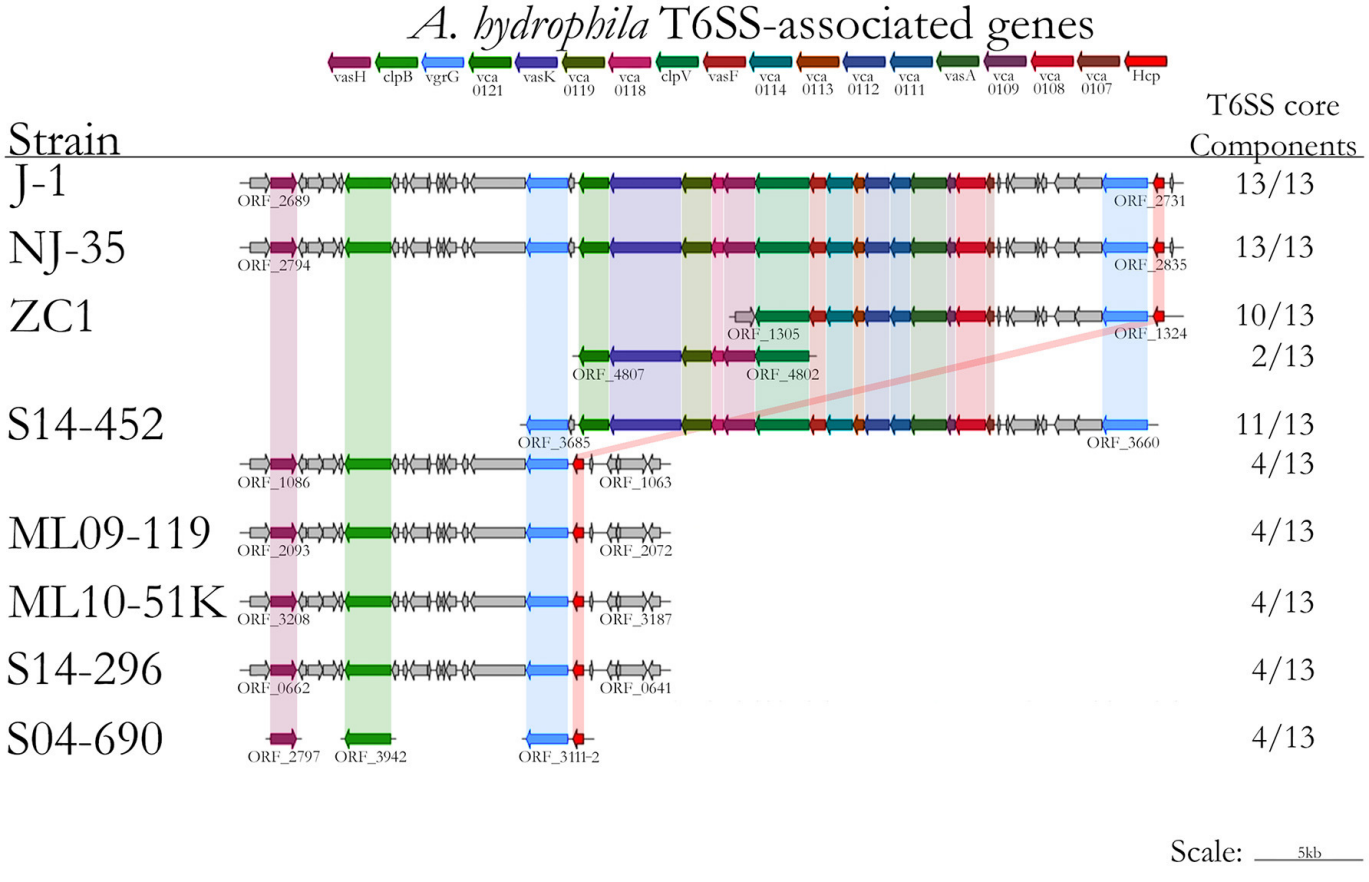
**Type VI secretion system gene prediction using the T346 Secretion System Hunter, with results including strains included in the immersion catfish challenge (ML09-119, MNL10-51K, S04-690, S14-296, S14-452, and ZC1) and representatives from Chinese strains (J-1, NJ-35, and ZC1)**.

Conserved sequences from the core genome analysis were used to infer phylogenetic relationships among the 61 *Aeromonas* spp. isolates (Figure 1A). The core genome phylogeny indicates with 100% bootstrap support that vAh strains form a monophyletic group that is fundamentally distinct from other *A. hydrophila* (Figure 1A). In addition to supporting the monophyly of these ST251 isolates, this phylogenetic analysis also revealed support for distinct sub-clades among vAh isolates, with 2660 single nucleotide polymorphisms identified between the vAh sub-clades. For example, *A. hydrophila* ZC1, isolated from a grass carp in China (Figure 1B), is closely affiliated with vAh strains isolated from catfish in MS (S14-452, S14-458, S15-130, S15-400, and S15-591). A key genotype that differentiates the vAh sub-clades is the presence and genetic organization of T6SS components (Figure 2). Each of the ZC1-affiliated strains, as well as the other strains isolated from carp in China (i.e., J-1 and NJ-35), were found to contain at least 80% of the core proteins necessary for a complete T6SS, with ZC1 and S14-452 having two separate T6SS-associated gene clusters, whereas J-1 and NJ-35 each have a single complete T6SS cluster (Figure 2). In contrast, vAh isolates from catfish in AL as well as other MS isolates (i.e., S13-612, S13-700, S14-606, and S14-296) formed a distinct subclade that lack the majority of the core T6SS genes (Figures 1B, 2). Notably, while AL and MS vAh strains lacked the majority of T6SS components, they consistently retain other genes that are involved in T6SS in other bacteria such as a valine-glycine repeat protein G (VgrG), a T4 bacteriophage tail-like hole forming protein (Leiman et al., 2009); hemolysin coregulated protein (Hcp), a repetitive tubular protein that is similar to the phage major tail protein GpV (Pell et al., 2009); the chaperone protein ClpB, a chaperone and ATPase that interacts with Hcp to translocate effectors (Shrivastava and Mande, 2008); and VasH, a putative transcriptional regulator (Kitaoka et al., 2011) (Figure 2).

### Average nucleotide identity

**Figure 3:**
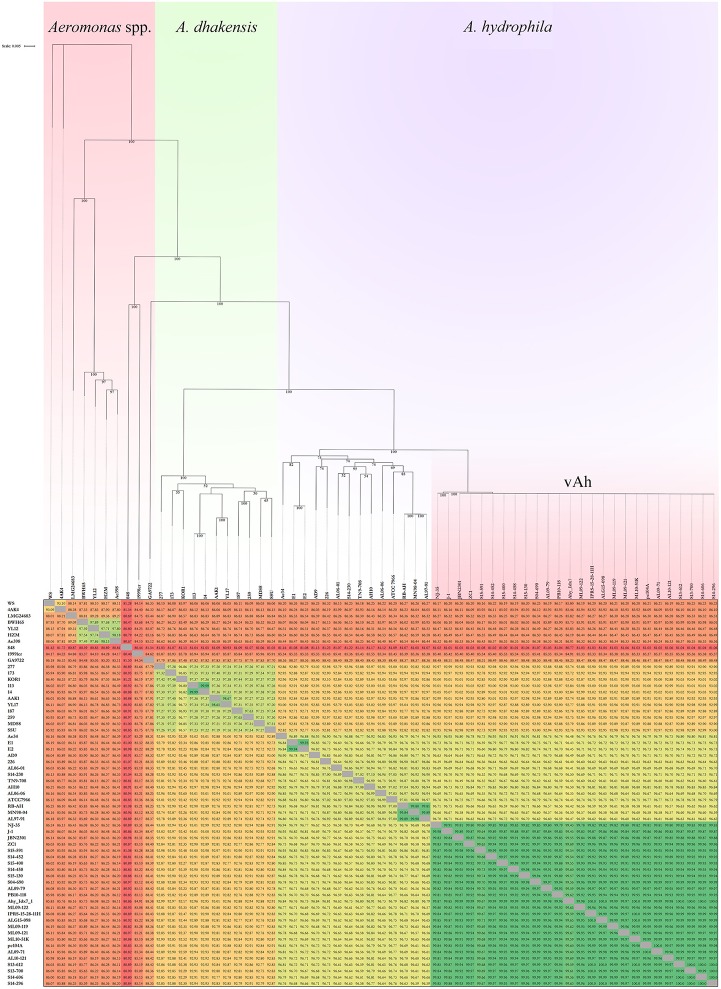
**Average nucleotide identities (ANI) among *Aeromonas* spp. strains and an associated maximum likelihood phylogram based on a core genome phylogeny (Figure 1)**. Please note that the branch length of strains 848 and 1999lcr were reduced to improve readability and pairwise ANI-values are color-coded according to percent identity.

The average nucleotide identity values of the 61 *Aeromonas* spp. genomes was determined (Figure 3). High ANI-values (>99%) were found for all vAh-vAh pairwise comparisons, supporting the core genome phylogeny (Figures 1B, 3). In contrast, all vAh comparisons with non-vAh isolates possessed ANI-values less than 97%. In accordance with previous results, 14 strains appear to have a discrepancy between the species classification listed in GenBank and the species affiliation indicated by ANI-values (Table 1).

### Virulence of vAh strains in channel catfish using an immersion challenge model

**Figure 4:**
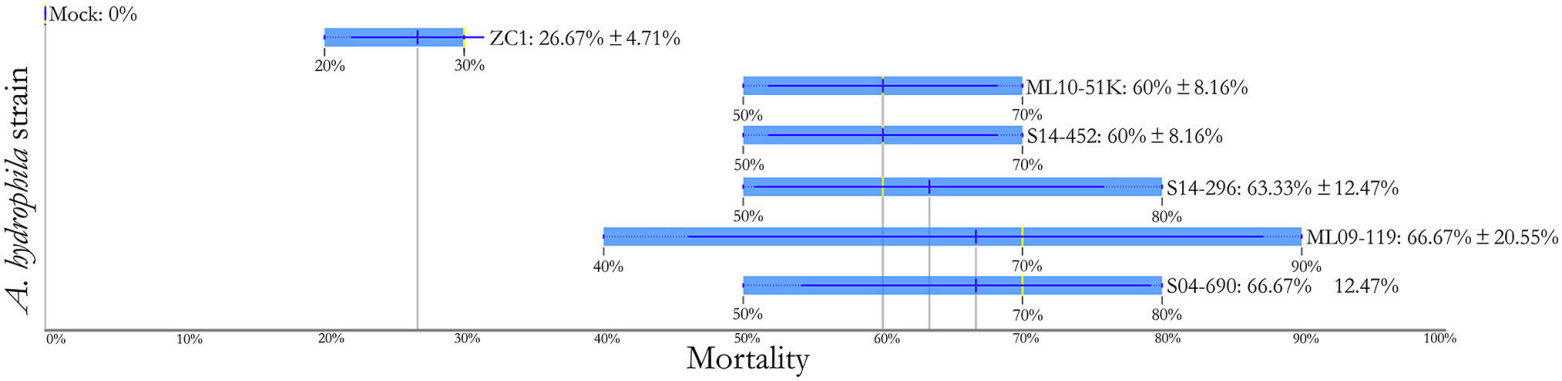
**Comparative assessment of the relative virulence of vAh isolates in channel catfish using 1 h immersion exposure with fin clip (ANOVA = 7.628, *p*-value = 0.001)**.

Challenge of channel catfish with vAh isolates from AL and MS resulted in ≥ 60% mortality (Figure 4). Within these US vAh isolates tested, there were no observed differences in virulence among ML09-119, ML10-51K, S04-690, S14-296, and S14-452, based on Duncan's multiple range test (*p*-value > 0.05). The carp isolate ZC1 was less virulent than AL and MS isolates with only 26.7% mortality observed (Figure 4). Most mortality (~96%) occurred within 48 h post challenge for all isolates, including ZC1. All dead fish (100%) sampled for confirmation were positive for the presence of vAh in liver tissue. Control fish yielded no mortality from the mock challenge.

### Pathology of epidemic *A. hydrophila* infections

**Figure 5:**
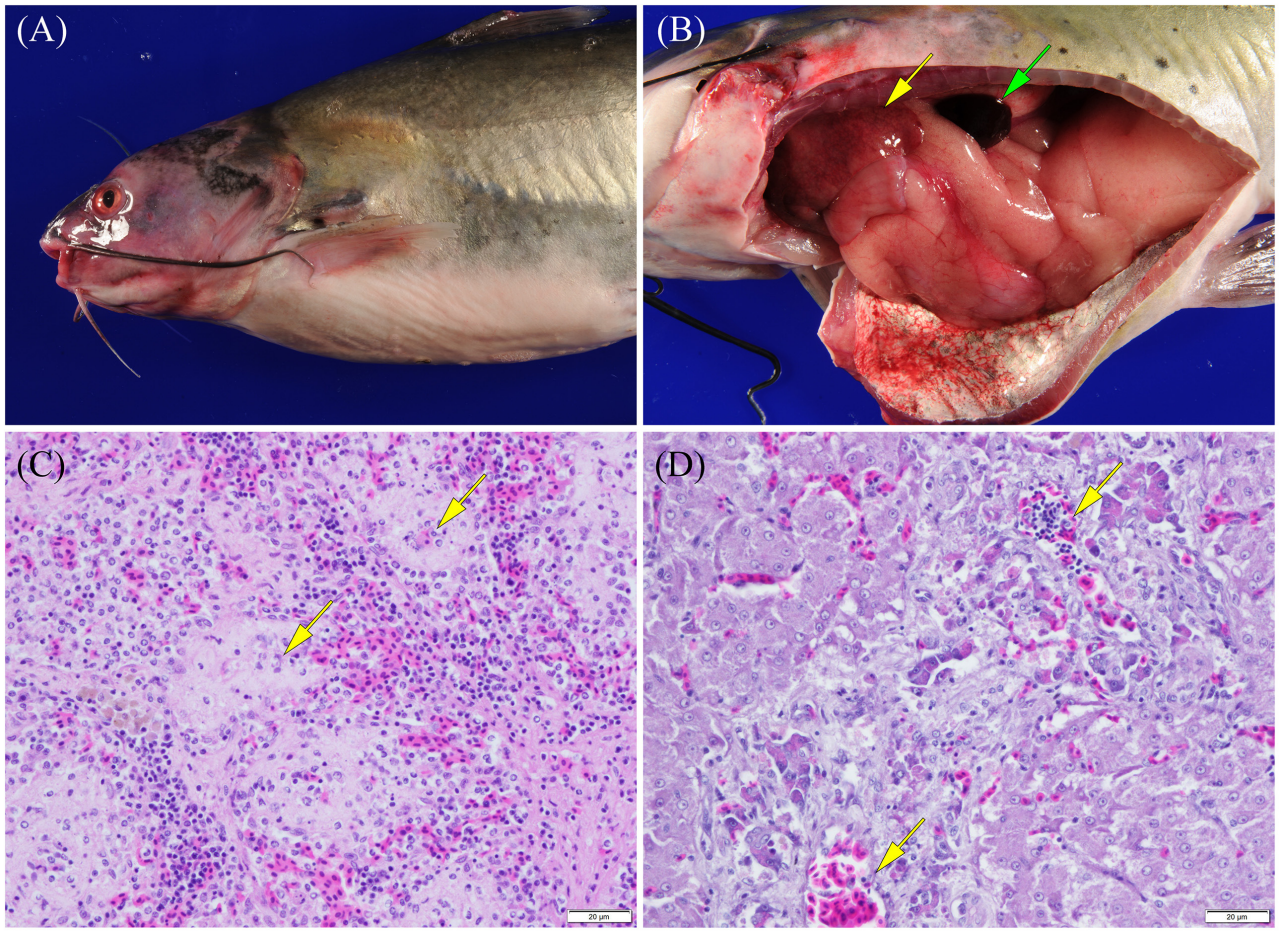
**Photographs of channel catfish infected by vAh showing (A) external surfaces that are exhibiting congestion/hemorrhage around the head/pectoral fin and within the eye and (B) the celomic cavity that has internal organs moderately congested and enlarged, a congested/hemorrhagic spleen (green arrow), and multifocal pale foci corresponding to areas of necrosis (yellow arrow) scattered over the liver (photographs courtesy of Dr. Wes Baumgartner, Mississippi State University) as well as photomicrographs of a channel catfish infected by vAh strain ML09-119 showing (C) a section of spleen with splenic ellipsoids (arrows) that are edematous and ellipsoidal arteries that are lined by degenerating as well as necrotic endothelial cells and (D) a section of liver with edema and necrosis of pancreatic acinar tissue surrounding branches of the hepatic portal vein (arrows)**.

Cutaneous lesions observed in fish infected with vAh by immersion challenge included extensive hyperemia over the pale ventrum of the fish, in tissues surrounding and within the mouth and at fin bases (Figure 5A). There was also extensive hyperemia around the eyes and exophthalmos in some fish. Cutting into the muscle of the lateral body wall revealed multifocal to coalescing foci of congestion/hemorrhage. Gill lesions were variable; gills were pale in some fish and reddened in others. Internally, there was widespread hyperemia of abdominal organs as well as petechial and ecchymotic hemorrhages scattered over mesenteric tissues. The spleen was moderately to severely swollen and dark red (Figure 5B). Head and trunk kidneys were moderately edematous and red and friable when harvested. The intestinal tract was mildly to moderately dilated and red (Figure 5B). The liver was mildly to moderately swollen with slightly rounded edges (Figure 5B). Glisson's capsule was peppered with variable numbers of petechial and ecchymotic hemorrhages. The atrial chamber of the heart was dilated and filled with blood.

Histopathologic lesions observed were strongly suggestive of a septic disease and included edema and necrosis in many internal organs. Splenic lesions included ellipsoidal necrosis and congestion/hemorrhage in the splenic red pulp (Figure 5C). In scattered necrotic ellipsoids variable numbers of short, rod-shaped bacteria were observed. In the liver there was often necrosis of the acinar pancreatic tissue surrounding hepatic vessels (Figure 5D). Scattered acinar cells were rounded and necrotic and extracellular zymogen granules could be observed in the necrotic exudate. Small numbers of inflammatory cells including macrophages, lymphocytes, and neutrophils were observable in scattered areas of pancreatic acinar necrosis. Multifocally within the hepatic parenchyma were variable sized foci of hepatocellular necrosis characterized by the presence of small aggregates of degenerating and necrotic hepatocytes. Hematopoietic cells in the renal interstitum and renal epithelial cells lining scattered renal tubules were undergoing degeneration and necrosis. In sections of head kidney, the tissue was edematous and congested/hemorrhagic. There was scattered degeneration and necrosis of red and white cell precursors. Intestinal lesions were minimal in fish infected in this fin clip model and were limited to mild congestion and hemorrhage of the vessels in the lamina propria and vessels of the muscularis and serosa. In some sections of heart there was mild necrosis of myofibers in the myocardium. Vessel of the brain were often moderately dilated and congested but the neuropil of the cerebrum, cerebellum, and brain stem was normal. The epithelium of the gills was normal but branchial capillaries were sometimes dilated and congested with erythrocytes.

### Differentiating pathotypes

**Figure 6:**
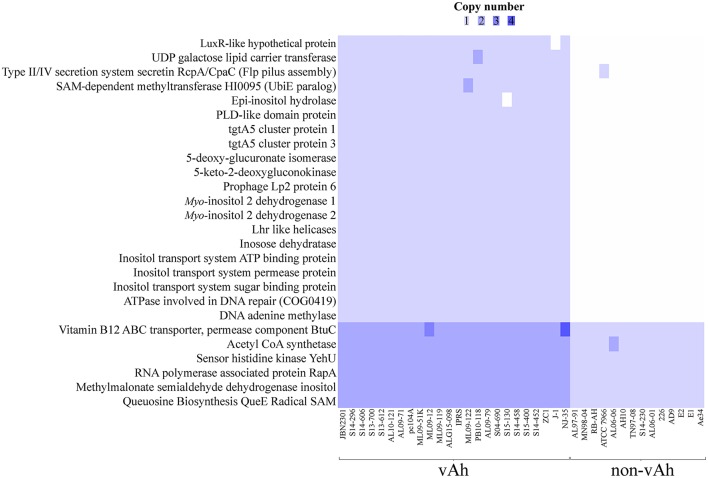
**Comparative whole genome predicted gene-based analysis of all confirmed vAh (*n* = 26) and non-vAh isolates (*n* = 15)**.

In order to robustly define the vAh pathotype-specific loci in this study, previous results on established *A. hydrophila* virulence factors as well as genes unique to vAh strains were used in combination with a clustering approach and a random forest decision tree to identify gene products that may contribute to functional differences in virulence. This approach confirmed previously described gene clusters for L-fucose and O-antigen biosynthesis as well as *myo*-inositol catabolism. Additionally, we identified predicted virulence factors conserved within all vAh strains (Table 3) which includes virulence factors well-known to be important in *A. hydrophila* pathogenesis (Rasmussen-Ivey et al., 2016). Among predicted virulence factors conserved among vAh strains, there were many genes uniquely associated with vAh strains that were not present in other sequenced *A. hydrophila* strains, including a L-serine dehydratase, a N-acetylmannosamine kinase, a N-acetylneuraminate lyase, and a gene product predicted to be required for queuosine biosynthesis (Table 4). In a more comprehensive approach, all RAST/SEED predicted genes from the 41 confirmed *A. hydrophila* genomes (26 vAh and 15 non-vAh) included in this study were evaluated on the basis of linkage with the vAh pathotype using exhaustive iterations of random forest modeling, which resulted in 26 genes uniquely associated with vAh by either presence/absence or by differential copy number when compared to non-vAh (Figure 6).

**Table 3:** **Predicted virulence factors that are conserved within vAh strains (not unique to), based on a comparison of significant BLASTn hits between vAh isolates against the VFDB (virulence factors with additional results are marked with an asterisk and are available in supplementary data)**.

**Putative virulence factor**	**Gene**	**Uniprot ID**	**GI**	**Reference bacterium**
3-oxoacyl-acyl carrier protein synthase II	*fabB*	A0A0H2V610	77416726	*Escherichia coli* O6:H1
Acriflavine resistance protein AcrB	*acrB*	P31224	25009252	*Escherichia coli* K12
Aerolysin/hemolysin/cytolytic enterotoxin	*ahh*	Q06303	89276735	*Aeromonas hydrophila* AH-1
Asparaginyl-tRNA synthetase	*asnS*	Q56112	16502162	*Salmonella enterica* serovar Typhi CT18
Cephalosporinase; class C beta lactamase	*ampC*	Q8KU09	21311545	*Aeromonas caviae* CIP 74.32
Enterochelin esterase	*fes*	A0A0H2V760	26106962	*Escherichia coli* O6:H1
Ethanolamine utilization protein EutN	*eutN*	B7LTU3	984388511	*Escherichia fergusonii* ATCC 35469
Flagellar motor switch protein FliN	*fliN*	A0A0H3QVI1	674744044	*Pseudomonas aeruginosa* Stone 130
General secretion pathway protein PulF	*pulF*	P15745	149305	*Klebsiella pneuoniae oxytoca* UNF5023
Protein translocase subunit SecA	*secA*	Q8YJG2	672757090	*Brucella melitensis* biotype 1
Rod shape-determining protein MreB	*mreB*	P0A9X4	557273544	*Escherichia coli* K12
Sodium/proline symporter proline permease	*putP*	P07117	131658	*Escherichia coli* K12
Transcriptional activator NtrC	*ntrC*	O86057	5731350	*Herbaspirillum seropedicae* DCP286A
Twitching motility protein PilU	*pilU*	G3XCX3	15595593	*Pseudomonas aeruginosa* PAO1

**Table 4:** **Virulence factors that are unique to vAh strains, based on a comparison of significant BLASTn hits between *A. hydrophila* isolates against the RAST/SEED database**.

**Subsystem**	**Role**	**GI**
Glycine/serine Utilization	L-serine dehydratase	958619257
Inositol catabolism	5-deoxy-glucuronate isomerase	958618826
Inositol catabolism	5-keto-2-deoxygluconokinase	827371814
Inositol catabolism	Epi-inositol hydrolase	612156152
Inositol catabolism	Inositol transport system ATP-binding protein	958621246
Inositol catabolism	Inositol transport system permease protein	958620586
Inositol catabolism	Inositol transport system sugar-binding protein	657060685
Inositol catabolism	Inosose dehydratase	507222178
Inositol catabolism	*Myo*-inositol 2-dehydrogenase 1	656991783
Inositol catabolism	*Myo*-inositol 2-dehydrogenase 2	827371809
Inositol catabolism	Transcriptional regulator of the *myo*-inositol catabolic operon	958618669
Sialic Acid Metabolism	N-acetylmannosamine kinase	827373367
Sialic Acid Metabolism	N-acetylneuraminate lyase	1043232173
Sialic Acid Metabolism	Predicted sialic acid transporter	446588390
Sialic Acid Metabolism	Sugar isomerase involved in processing of sialic acid	958620857
Pyridoxin Biosynthesis	Predicted transcriptional regulator of pyridoxine metabolism	16078013
Phage DNA synthesis	DNA adenine methyltransferase, phage-associated	67483065
Phage capsid proteins	Phage capsid scaffolding protein	516389014
Phage capsid proteins	Phage major capsid protein	507220251
Phage lysis modules	Phage lysin, 1,4-beta-N-acetylmuramidase	511291760
Phage packaging machinery	Phage portal protein	958618794
Phage packaging machinery	Phage terminase small subunit	759443491
Phage packaging machinery	Phage terminase, large subunit	958620694
Queuosine-Archaeosine Biosynthesis	Queuosine biosynthesis QueD, PTPS-I	1043232409
Queuosine-Archaeosine Biosynthesis	archaeosine tRNA-ribosyltransferase type 5	507221161

## Discussion

The core genome analysis of epidemic *A. hydrophila* strains obtained from diseased fish from several US states and Chinese provinces (identified within the literature as ST251/vAh), supports the genetic and functional unification of these hypervirulent bacteria within a monophyletic clade. These data are in agreement with previous reports based on single or multiple genetic loci from smaller numbers of vAh strains (Hossain et al., 2014; Pang et al., 2015). Interestingly, this study found evidence for genomic heterogeneity among the sampled vAh strains that may reflect geographic origin and/or host switching. For example, the isolates obtained from MS in 2013–2015 are affiliated with two different ST251/vAh clades, specifically the Asian carp-affiliated clade and the US catfish-affiliated clade. In contrast, the vAh isolates from diseased catfish in AL from 2009 to 2015 reflect a single, clonal clade. These data suggest that vAh has greater diversity within MS aquaculture ponds compared to those of AL. As previously hypothesized, this pattern would fit with a dissemination model in which carp or other fish or fish products from Asian source(s) were first introduced to the Mississippi delta region, after which a particularly more virulent vAh lineage spread among farmed catfish resulting in the initial epidemic outbreaks within AL.

Genomic comparisons indicate all members of the vAh pathotype strains share unique genetic loci that may be a result of their close genetic affiliation and may also contribute to their pathogenesis. The previously identified vAh-specific gene clusters of L-fucose and *myo*-inositol catabolism were also confirmed in this study. The use of *myo-*inositol as a sole carbon source is a rarely observed phenotype among *Aeromonas* species and, to our knowledge, has only been reported in vAh strains and strains of *A. finlandiensis* (Beaz-Hidalgo et al., 2015b). This study identified additional genetic loci that are present in all sequenced vAh strains and may have a contribution to virulence, such as a L-serine dehydratase, a N-acetylmannosamine kinase, a N-acetylneuraminate lyase, a sialic acid transporter, a transcriptional regulator of pyridoxine metabolism, an archaeosine tRNA-ribosyltransferase, a gene product required for queuosine biosynthesis (QueD), an acriflavine resistance protein A, and an IS5 transposase and transactivator. While the contribution of each of these gene products to vAh virulence has yet to be determined, studies in other bacterial pathogens suggest potential mechanisms that could enhance vAh virulence. For example, in *Campylobacter jejuni* a L-serine dehydratase is essential for gut colonization (Velayudhan et al., 2004). With regard to sialic acid, *Vibrio cholera* has been shown to use this system to evade the innate immune response (Severi et al., 2007), and promote the binding of cholera toxin to the host intestinal epithelium (Rohmer et al., 2011). In addition, increased acriflavine resistance may provide a selective advantage to vAh considering that acriflavine is a commonly used antiseptic in aquaculture (Martin, 1968).

All annotated genes were subsequently evaluated for linkage with the vAh genotypes, a method that removes the bias inherent in assuming that only known virulence factors contribute to vAh pathogenesis. This analysis revealed that 26 genes are synonymous with the vAh pathotype either by presence/absence or by copy number. Supporting the robust nature of this approach, previously described genes that are vAh-associated were also identified, including genes associated with the *myo-*inositol catabolic pathway. Of note, within this pathway a methylmalonate-semialdehyde dehydrogenase that is required for *myo-*inositol catabolism was identified in both vAh and non-vAh isolates; however, this enzyme is required for both *myo*-inositol and valine metabolism and there is no indication that non-vAh strains have the genetic capacity for *myo-*inositol catabolism. In addition to these results, this method also identified known *Aeromonas* spp. virulence factors, such as a DNA adenine methylase that has been identified as a regulator of virulence and required for viability in *A. dhakensis* SSU (Erova et al., 2006). From this approach other putative vAh virulence factors were identified, such as a hypothetical protein with a LuxR-like domain. LuxR has been previously shown to be a regulator of virulence factors and quorum sensing within *Aeromonas* spp. (Kirke et al., 2004). Lastly, other genetic loci were identified as being associated with vAh strains, such as the type II/IV system secretin RcpA/CpaC that is putatively involved in flp pilus assembly (Clock et al., 2008), but to our knowledge the contribution of this secretin to *A. hydrophila* pathogenesis has yet to be experimentally determined.

The histopathologic examination of tissues from vAh-infected farmed fish show a wide range of severity related to internal lesions, with some fish exhibiting minimal lesions and others having widespread sepsis with necrosis of spleen, liver, renal tissue, intestine, and brain tissues with subsequent high rates of mortality occurring throughout affected farms (W. Hemstreet, personal communication). For fish challenged with vAh strains in aquaria via intraperitoneal injection, rapid onset of mortality without these disease sequelae was observed (Hossain et al., 2014). In this study, fish were challenged with an immersion model and exhibited significant clinical signs including cutaneous and ocular hemorrhaging, splenic and renal congestion, and hemorrhage with mild to moderate necrosis of internal organs. Despite the high genetic similarity of the strains (ANIs > 99%), strain ZC1 had reduced virulence (~27% mortality) when compared with ML09-119; ML10-51K; S04-690; S14-296; and S14-452 which caused ≥ 60% mortality in channel catfish. The reduced virulence of strain ZC1, relative to strain ML09-119, in carp and in catfish was previously observed when fish were challenged intraperitoneally (Hossain et al., 2014). Interestingly, comparatively few reports of mortalities due to MAS have come from the state of MS, which may reflect a number of geographic differences, the heterogeneity of vAh strains present within MS aquaculture ponds, differences in management and production practices, and/or environmental conditions.

To identify genetic elements that may explain the disparity in virulence between the Asian carp isolate ZC1 and the US catfish isolates, genomic comparisons were conducted between ZC1 and the vAh strains included in the immersion challenge. These analyses revealed that all other vAh isolates tested in the disease challenge, but not strain ZC1, encode two putative transcription regulators: (1) a mobile element protein in the helix-turn-helix (HTH) superfamily and (2) a phage antirepressor protein in the *antA* superfamily present in *Escherichia coli* ECOR-9 (Sandt et al., 2002). The presence of these transcription factors within the highly virulent vAh strains could result in increased virulence factor expression in these strains. Furthermore, vAh isolates from Asian carp or the ZC1-affiliated clade from MS (including S14-452) were found to have a potentially functional T6SS, but the clade comprised solely of AL and MS catfish isolates (e.g., ML09-119, ML10-51K, S04-690, and S14-296) were found to consistently lack 9 of 13 core T6SS genes related to secretion of virulence factors such as Hcp. The observation that the isolates in the AL/MS vAh subclade lack a complete and/or functional T6SS would lead one to hypothesize that virulence is attenuated in these vAh strains; however, our experimental results indicate that these AL/MS disease isolates are highly virulent compared to strain ZC1. Previous disease challenges with strain ZC1 using intraperitoneal injection support the conclusion that this Asian carp strain ZC1 has reduced virulence relative to the AL/MS vAh clade in both catfish and carp (Hossain et al., 2014), indicating that the attenuated virulence observed in strain ZC1 is not solely attributable to host-specific differences. The vAh strains in the AL/MS subclade encode only a subset of T6SS components that include VgrG, Hcp, ClpB, and VasH. The absence of the other T6SS core genes, including *vca0107-0109, vca0111-0114, vca0118, vca0119, vca0121, vasK, clpV, vasF*, and *vasA*, largely accounts for the ~16 Kbp net difference in the genome sizes between strains ZC1 and ML09-119. Based on studies of T6SS in *Aeromonas dhakensis* SSU and in *Salmonella enterica* serovar Typhi (Suarez et al., 2008; Wang et al., 2011), it is hypothesized that US isolates lacking several T6SS proteins would exhibit better evasion of the host's immune response. Future research should aim to elucidate the role of these T6SS components, and the effect of a reduced or rearranged T6SS, on vAh virulence.

As new vAh isolates emerge and our collective knowledge of vAh genomic diversity grows, future research should investigate the spread of these pathogens as well as improve the design of more effective biosecurity strategies for the aquaculture industry. Toward this effort, we report a primer set that differentiates vAh from non-vAh strains and is inclusive of the known diversity of vAh strains in the US and Asia. The combination of tools available to differentiate vAh strains from non-vAh strains, including qPCR and the *myo*-inositol growth assay, are vital tools that can be used to map the global distribution of vAh in carp, catfish, and other warm-water fish species (e.g., tilapia). Disease control strategies should take into account the variability observed in this study among vAh strains and evaluate the efficacy of vaccination or other control measures against a panel of strains that represent the known diversity of this highly virulent *A. hydrophila* pathotype.

In conclusion, hypervirulent *A. hydrophila* within ST251 have emerged as pathogens of farmed warmwater fishes that are classified within the vAh pathotype based on strong phylogenetic evidence that includes a core genome phylogeny and ANI-values >99%; metabolic activities that are unique within this species, such as *myo*-inositol and sialic acid metabolism; a suite of conserved *Aeromonas* spp. virulence factors; 26 conserved genetic loci putatively linked with virulence; and the ability to induce motile *Aeromonas* septicemia, which is characteristically followed by rapid mortality in multiple species of farmed fish. Collectively, these traits distinguish vAh from non-epidemic *A. hydrophila* and define the vAh pathotype.

## Author contributions

CR generated and analyzed genome sequence data; CR and SO analyzed gene copy number for putative virulence factors; JT and WH isolated vAh disease isolates from AL and collected data on fish disease; CS, DZ, and DX conducted vAh disease trials; CR and MH developed vAh-specific primer sets; MG collected disease isolates from Manuscript and typed them using different primer sets and bioassays, YL collected disease isolates from Chinese carp and analyzed data, MF contributed to the phylogenetic analysis and inositol utilization of *Aeromonas* isolates, SS conducted the core genome phylogenetic analysis, JN conducted the histology analysis and contributed to the writing and editing of the manuscript; and ML contributed to the genome sequence analysis, organizing the different research activities and to the writing and editing of the manuscript.

## Funding

This work was supported by a grant from the United States Department of Agriculture's Agriculture and Food Research Initiative (Projects #2013-67015-21313 and #MIS-371530) and the USDA-ARS (CRIS Project No. 6010-32000-026-00D and the Catfish Health Initiative). MF thanks the support by the projects: Aquavalens from the European Union Seventh Framework Program (FP7/2007-2013) under Grant agreement No.: 311846, and by projects from Spanish Ministry of Science and Innovation: AGL2011-30461-C02-02 and JPIW2013-095-CO3.

### Conflict of interest statement

The authors declare that the research was conducted in the absence of any commercial or financial relationships that could be construed as a potential conflict of interest.
