# Supplementary material for: Classification of a Hypervirulent Aeromonas hydrophila Pathotype Responsible for Epidemic Outbreaks in Warm-Water Fishes
Source: Front Microbiol. 2016 Oct 18;7:1615. doi: 10.3389/fmicb.2016.01615 (PMC5067525; doi:10.3389/fmicb.2016.01615)
Supplement: Supplementary file 1 [file Table1.docx]

**Supplemental Table 1.** Sequence statistics of 14 sequenced vAh genomes.

| **vAh strain** | **Genome size (Mbp)** | **Percent G+C** | **Average coverage** | **Average read length (bp)** | **Average contig length (kbp)** | **NCBI accession** |
| --- | --- | --- | --- | --- | --- | --- |
| Ahy_Idx71 | 3.30 | 61.8 | 118.99 | 97.06 | 8.895 | [SAMN05292361](http://www.ncbi.nlm.nih.gov/biosample/5292361) |
| ALG15-098 | 4.94 | 60.9 | 56.82 | 131.49 | 115.613 | [SAMN05223361](http://www.ncbi.nlm.nih.gov/biosample/?term=aeromonas+alg15-098) |
| IPRS15-28 | 4.95 | 60.9 | 51.77 | 129.99 | 110.519 | [SAMN05223362](http://www.ncbi.nlm.nih.gov/biosample/?term=aeromonas+iprs) |
| ML10-51K | 4.94 | 60.9 | 40.97 | 110.22 | 103.564 | [SAMN05223363](http://www.ncbi.nlm.nih.gov/biosample/?term=aeromonas+ml10%3D51k) |
| S13-612 | 4.96 | 60.9 | 26.76 | 114.32 | 40.696 | [SAMN05292362](http://www.ncbi.nlm.nih.gov/biosample/?term=aeromonas+s13-612) |
| S13-700 | 4.97 | 60.9 | 30.89 | 115.69 | 56.427 | [SAMN05292363](http://www.ncbi.nlm.nih.gov/biosample/?term=aeromonas+s13-700) |
| S14-230 | 4.70 | 61.4 | 26.52 | 120.53 | 45.667 | [SAMN05292364](http://www.ncbi.nlm.nih.gov/biosample/?term=aeromonas+s14-230) |
| S14-296 | 4.96 | 60.9 | 28.99 | 122.95 | 56.416 | [SAMN05292365](http://www.ncbi.nlm.nih.gov/biosample/?term=aeromonas+s14-296) |
| S14-458 | 5.00 | 60.8 | 53.40 | 132.62 | 93.109 | [SAMN05223364](http://www.ncbi.nlm.nih.gov/biosample/?term=aeromonas+s14-458) |
| S14-606 | 4.97 | 60.9 | 21.87 | 123.14 | 37.618 | [SAMN05292366](http://www.ncbi.nlm.nih.gov/biosample/?term=aeromonas+s14-606) |
| S15-130 | 4.86 | 61.2 | 33.37 | 135.6 | 36.641 | [SAMN05223365](http://www.ncbi.nlm.nih.gov/biosample/?term=aeromonas+s15-130) |
| S15-242 | 4.79 | 60.8 | 50.98 | 135.7 | 72.715 | [SAMN05223366](http://www.ncbi.nlm.nih.gov/biosample/?term=aeromonas+hydrophila+s15-242) |
| S15-400 | 4.97 | 60.9 | 72.64 | 112.42 | 75.075 | [SAMN05223367](http://www.ncbi.nlm.nih.gov/biosample/?term=aeromonas+s15-400) |
| S15-591 | 3.84 | 62.1 | 23.42 | 90.65 | 7.100 | [SAMN05223368](http://www.ncbi.nlm.nih.gov/biosample/?term=aeromonas+s15-591) |
